# *Streptococcus suis* Meningitis, United States

**DOI:** 10.3201/eid1401.070930

**Published:** 2008-01

**Authors:** Gregory T. Lee, Charles Y. Chiu, Barbara L. Haller, Patricia M. Denn, Christopher S. Hall, Julie L. Gerberding

**Affiliations:** * University of California San Francisco, San Francisco, California, USA; † San Francisco General Hospital, San Francisco, California, USA; ‡ Centers for Disease Control and Prevention, Atlanta, Georgia, USA

**Keywords:** Streptococci, *Streptococcus suis*, meningitis, letter

## Abstract

*Streptococcus suis* Meningitis, United States

**To the Editor:** *Streptococcus suis,* commensal and opportunistic pathogens of swine, and prevalent zoonotic agents worldwide, are α-hemolytic gram-positive cocci with 35 different serotypes (*1*). In humans, *S. suis* infection has been associated with bacterial meningitis, septic shock, arthritis, pneumonia, endocarditis, endophthalmitis, and spontaneous bacterial peritonitis (*2**,**3*). Most at risk are those who handle or eat undercooked pork, e.g., farm workers, butchers, and slaughterhouse workers (*4*). Most cases have been reported in Europe or Southeast Asia (*2**,**3*). Meningitis, first recognized in 1968 in Denmark (*1*), is the most common clinical manifestation of human infection with *S. suis*. A case of *S. suis* meningitis in a pig farmer was reported in the United States (*5*). Here, we describe another case in a 60-year-old man from San Francisco who had consumed raw pork while traveling in the Philippines.

In June 2003, this man became ill with fever, diaphoresis, headache, nausea, and anorexia. He had just returned from a 7-month vacation in the Philippines. Three days after symptoms onset, his physician prescribed doxycycline. Symptoms continued and he was admitted to a local hospital 5 days later with a fever of 38.9°C, nuchal rigidity, headache, and general malaise.

The patient described no recent contact with sick persons; past medical history was unremarkable. On physical examination, he was somnolent but fully oriented, with no focal findings on neurologic examination and only slight nuchal rigidity. He had a leukocyte count of 21,000/mm^3^, including 16,400/mm^3^ neutrophils. Cerebrospinal fluid (CSF) showed leukocyte count of 487/μL with 80% polymorphonuclear cells and 18% lymphocytes, and glucose and protein levels <20 mg/dL and <167 mg/dL, respectively. Gram stain of CSF showed gram-positive cocci in pairs (Figure). Empiric therapy (ceftriaxone, vancomycin, and ampicillin) for bacterial meningitis was begun. Computed tomographic scan of the head showed only sinusitis; findings of chest radiograph and transesophageal echocardiogram were negative.

**Figure:**
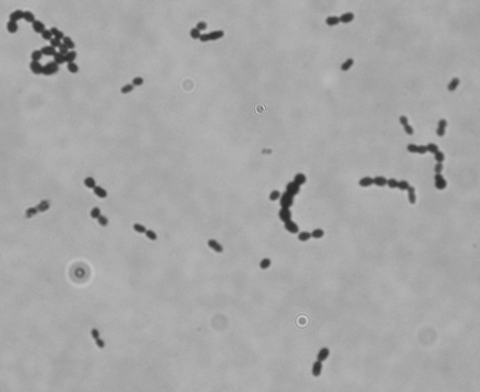
Gram-positive cocci in pairs in a 60-year-old man with meningitis. Magnification ×1,000.

On hospital day 2, blood cultures grew gram-positive cocci in pairs and chains. The organism was catalase-negative, bile esculin-negative, and pyrrolidonyl aminopeptidase-negative, consistent with *Streptococcus* spp. A latex agglutination test did not detect *Streptococcus pneumoniae* antigen. Antimicrobial susceptibility testing showed that the isolate was sensitive to penicillin (MIC = 0.03), ceftriaxone, and vancomycin but resistant to tetracycline and clindamycin. Antimicrobial therapy was changed to penicillin G, 24 million units intravenously per day.

On hospital day 5, the patient complained of hearing loss in his left ear. Results of nasopharyngeal endoscopy were negative. By hospital day 7, the organism was identified by the API 20 Strep System (bioMerieux, Marcy l’Etoile, France), as S*. suis* serotype 2. The patient subsequently stated that he was a butcher with a culinary preference for partially cooked pork, which he had eaten in the Philippines until the week prior to onset of symptoms. On hospital day 9, a formal audiology evaluation showed severe bilateral sensorineural high-frequency hearing loss (–70 dB). The patient completed a 10-day course of parenteral antimicrobial drugs and was discharged on continued oral therapy with close followup. Two months after discharge, the patient reported much improved hearing without other sequelae.

Most *S. suis* infections occur in older men and patients who report contact with pigs or eating undercooked pork products. Invasion of the bloodstream can occur directly through skin abrasions or the oral or respiratory route (*6*). Once bloodborne, *S. suis* can cause toxic shock syndrome and sepsis (*7*). The mechanism by which the organism traverses the blood-brain barrier to cause meningitis is not known, although bacterial toxins and host inflammatory mediators may play a role (*8*).

Hearing loss from *S. suis* meningitis, although not specific for the organism, occurs frequently in half to two thirds of patients and can be irreversible (*3**,**7**,**9*). Administering dexamethasone may ameliorate hearing loss in some cases (*10*). Penicillin G is the preferred treatment for *S. suis* infection, although penicillin resistance has emerged in *S. suis* because of the farm practice of supplementing feeds with antimicrobial drugs. As an alternative therapy, vancomycin may be used (*6*). Thus, empiric therapy for adult bacterial meningitis (ceftriaxone and vancomycin with or without ampicillin) would likely be sufficient to treat *S. suis* meningitis. Although the death rate from this disease can be high, varying from 7% in one study (*3*) to 30% in another (*6*), infection can be prevented by treating abrasions promptly, wearing gloves when handling pork, adhering to proper hand washing techniques, and sufficiently cooking pork products (*3*).

*S. suis* infection may go unrecognized since many laboratories do not routinely speciate α-hemolytic streptococci. However, in the United States, specialized tests such as the API 20 Strep System (API System; La Balme Les Grottes, Montalieu-Vercieu, France) or reference laboratories are readily available for diagnosis of all unidentified streptococci*.* In severe cases where infection is suspected, physicians may request that laboratories conduct definitive tests to identify the organism. In countries that lack these resources and where undercooked pork is a diet staple, underdiagnosis of *S. suis* infection is likely. Greater understanding of this organism and its disease spectrum would promote earlier diagnosis and prevention of sequelae.
